# Potential involvement of lactate and interleukin-6 in the appetite-regulatory hormonal response to an acute exercise bout

**DOI:** 10.1152/japplphysiol.00218.2017

**Published:** 2017-07-06

**Authors:** Hashim Islam, Logan K. Townsend, Greg L. McKie, Philip J. Medeiros, Brendon J. Gurd, Tom J. Hazell

**Affiliations:** ^1^Department of Kinesiology and Physical Education, Wilfrid Laurier University, Waterloo, Ontario, Canada;; ^2^Department of Molecular and Cellular Biology, University of Guelph, Guelph, Ontario, Canada; and; ^3^School of Kinesiology and Health Studies, Queen’s University, Kingston, Ontario, Canada

**Keywords:** appetite regulation, gut peptides, orexigenic, anorexigenic, energy intake

## Abstract

High-intensity exercise suppresses appetite partly through changes in peripheral appetite-regulating hormones. Lactate and IL-6 mediate the release of these hormones in animal/cell models and may provide a mechanistic link between exercise intensity and appetite regulation. The current study examined changes in appetite-regulating hormones, lactate, and IL-6 after different intensities of running. Eight males completed four experimental sessions: *1*) moderate-intensity continuous training (MICT; 65% V̇o_2max_); *2*) vigorous-intensity continuous training (VICT; 85% V̇o_2max_); *3*) sprint interval training (SIT; repeated “all-out” sprints); and *4*) Control (CTRL; no exercise). Acylated ghrelin, active glucagon-like peptide-1 (GLP-1), total peptide YY (PYY), lactate, IL-6, and appetite perceptions were measured pre-, immediately postexercise, 30 min postexercise, and 90 min postexercise. Energy intake was recorded over 3 days. VICT and SIT suppressed ghrelin (*P* < 0.001), although SIT elicited a greater (*P* = 0.016 vs. MICT) and more prolonged (*P* < 0.001 vs. all sessions) response. GLP-1 increased immediately after MICT (*P* < 0.001) and 30 min after VICT (*P* < 0.001) and SIT (*P* < 0.002), while VICT elicited a greater postexercise increase in PYY vs. MICT (*P* = 0.027). Postexercise changes in blood lactate and IL-6 correlated with the area under the curve values for ghrelin (*r* = −0.60, *P* < 0.001) and GLP-1 (*r* = 0.42, *P* = 0.017), respectively. Appetite was suppressed after exercise (*P* < 0.001), although more so after VICT (*P* < 0.027) and SIT (*P* < 0.001) vs. MICT, and energy intake was reduced on the day after VICT (*P* < 0.017 vs. MICT and CTRL) and SIT (*P* = 0.049 vs. MICT). These findings support an intensity-dependent paradigm for appetite regulation following exercise and highlight the potential involvement of lactate and IL-6.

**NEW & NOTEWORTHY** This study examines the involvement of two potential mechanisms (lactate and IL-6) that may explain the intensity-dependent effects of acute exercise on appetite-related parameters. Our findings support a clear intensity-dependent paradigm for appetite regulation following exercise, as highlighted by the change in acylated ghrelin and the suppression of appetite and energy intake after vigorous exercise (continuous and intermittent). Further, our findings extend previous work in animal/cell models by providing evidence for the potential role of lactate and IL-6 in mediating changes in appetite-related parameters following exercise in humans.

## Abstract (precis)

This study examines the involvement of two potential mechanisms (lactate and IL-6) that may explain the intensity-dependent effects of acute exercise on appetite-related parameters. Our findings support a clear intensity-dependent paradigm for appetite-regulation following exercise, as highlighted by the change in acylated ghrelin and the suppression of appetite and energy intake after vigorous exercise (continuous and intermittent). Further, our findings extend previous work in animal/cell models by providing evidence for the potential role of lactate and IL-6 in mediating changes in appetite-related parameters following exercise in humans.

the physiological control of energy intake is mediated by complex interactions between key brain regions involved in energy homeostasis and circulating appetite-regulating hormones (29). Acute energy intake is influenced by several gut-derived hormones that respond to changes in energy status and alter perceptions of appetite through central neuronal processes (23). Ghrelin is the only known gut-derived hormone with orexigenic (appetite-stimulating) properties and is released from gastric cells during periods of energy deficit, such as fasting (35). Once secreted, ghrelin is converted to its biologically active (acylated) form by the enzyme ghrelin O-acyl transferase (60). Acting in opposition to ghrelin are the anorexigenic (appetite-inhibiting) products of intestinal L cells, peptide YY (PYY) and glucagon-like peptide-1 (GLP-1) (29). Active GLP-1 (GLP-1_7–36_ and GLP-1_7–37_) is rapidly degraded to the inactive GLP-1_9–36_ by the enzyme dipeptidyl peptidase-4 (DPP-IV), which also facilitates the conversion between PYY_1–36_ and PYY_3–36_ (28). Although both forms of PYY are biologically active, PYY_3–36_ exerts more potent anorexigenic effects (12) and represents the major circulating form of this peptide (4).

Acute exercise (30–90 min) suppresses acylated ghrelin and increases GLP-1 and PYY concentrations (total and active), leading to a transient reduction in appetite and/or energy intake (45, 46). Furthermore, this hormonal response and the associated decrease in appetite and/or energy intake appears to be more pronounced after higher intensities of exercise (≥70% V̇o_2max_), highlighting exercise intensity as a potentially important appetite-regulatory stimulus (8, 9, 13, 15, 24, 25, 33, 34, 42, 52, 57). Specifically, comparisons between different intensities of moderate-intensity continuous training (MICT, 50–75% V̇o_2max_), and between MICT and high-intensity/sprint interval training (HIIT/SIT) suggest that intense exercise promotes greater changes in circulating hormone (acylated ghrelin, total PYY, PYY_3–36_) concentrations and that these changes are in a direction that favors reductions in appetite and/or energy intake (1, 8, 13, 15, 24, 42, 52, 57). However, the mechanisms underlying the potentially greater effects of high-intensity exercise on circulating hormone concentrations are unclear.

Although several mechanisms may link exercise intensity to appetite regulation (25), we were particularly interested in lactate and IL-6, as both of these can increase in an intensity-dependent manner following an acute exercise bout (11, 21, 48). Importantly for appetite regulation, lactate binds to ghrelin-producing cells and inhibits their secretory function (17), while its administration (peripheral and central) decreases energy intake in both humans (47) and animals (10, 36, 41). Additionally, elevations in IL-6 coincide with reduced postexercise energy intake in humans (2) and systemic increases in IL-6 (via infusion or following exhaustive exercise) stimulate GLP-1 secretion from intestinal L cells, while also increasing the expression of PYY mRNA in these cells (16). IL-6 also influences appetite and energy intake via direct actions on key neuronal circuits involved in energy homeostasis (18, 43, 44, 51). Thus, lactate may be involved in the intensity-dependent suppression of acylated ghrelin following exercise, while IL-6 may be important for increasing GLP-1 and PYY concentrations. Taken together, it can be hypothesized that lactate and IL-6, both of which can increase in an intensity-dependent manner following an acute exercise bout, mediate the effects of exercise intensity on appetite and energy intake due to their ability to influence circulating hormone concentrations.

To test the hypothesis that lactate and IL-6 are involved in the appetite-regulatory response to acute exercise, we utilized exercise intensity as a method to increase systemic lactate and IL-6 and examined their association with postexercise changes in appetite-related parameters. Specifically, we examined changes in orexigenic (acylated ghrelin) and anorexigenic (active GLP-1 and total PYY) gut peptides, appetite perceptions, and energy intake following two doses of submaximal continuous running (i.e., 65 and 85% V̇o_2max_), as well as a supramaximal interval training protocol, to investigate their relationship with exercise-induced increases in lactate and IL-6.

## MATERIALS AND METHODS

### 

#### Participants.

Eight active young males volunteered to participate in this study. Participants were nonsmokers and healthy, as assessed by the PAR-Q+ health questionnaire. All participants were physically active (≤3 weekly exercise sessions), although none were involved in a systemic training program nor had they been for at least 6 mo before data collection. Participants were not taking any dietary supplements at the time of the study. Experimental details were fully explained to all participants, and all provided written informed consent before any data collection. This study was approved by the Research Ethics Board at Wilfrid Laurier University in accordance with the 1964 Declaration of Helsinki.

#### Study design.

All participants completed four experimental sessions (~3 h each) in a systematic rotational order using a counterbalanced Latin square design, with each session performed ≥1 wk apart. Experimental sessions consisted of one resting control session (CTRL; no exercise) and three exercise sessions involving running-based protocols: *1*) moderate-intensity continuous training (MICT, 65% V̇o_2max_); *2*) vigorous-intensity continuous training (VICT, 85% V̇o_2max_); and *3*) sprint interval training (SIT, brief bouts of “all-out” running interspersed with short rest periods). Blood samples and subjective appetite measures were obtained at several time points during each session. Participants were instructed to refrain from physical activity, alcohol, and caffeine for ≥24 h before each experimental session. Energy intake was recorded for a 3-day period (day before, day of, day after), and participants were asked to replicate their dietary intake for 24 h before each session.

#### Preexperimental procedures.

All participants completed one familiarization session (1 wk) before the first experimental session to introduce testing procedures/equipment and reduce any learning effects during subsequent sessions. During this session, participants also performed a graded exercise test to exhaustion on a motorized treadmill (4Front, Woodway, WI) for the determination of maximal oxygen consumption (V̇o_2max_). Oxygen consumption (V̇o_2_) and carbon dioxide production (V̇co_2_) were measured continuously using an online breath-by-breath gas collection system (MAX II; AEI Technologies, Pittsburgh, PA) that was calibrated using known gas concentrations and a 3-liter syringe for flow. Heart rate (HR) was recorded beat-to-beat using an integrated HR monitor (FT1; Polar Electro, Lachine, QC, Canada). After a 5-min treadmill warm-up (3.5 mph), each participant ran at a self-selected pace (5–7 mph) that was maintained throughout the test, with incremental increases in workload (2% grade) applied every 2 min until volitional fatigue. V̇o_2max_ was defined as the greatest 30-s average at which V̇o_2_ values plateaued (<1.35 ml·kg^−1^·min^−1^) despite increases in workload, or two of the following criteria: *1*) respiratory exchange ratio (RER) value >1.10; *2*) maximal HR [within 10 bpm of age-predicted maximum (220-age)] and/or *3*) voluntary exhaustion. Following a 5-min treadmill cool-down and a short rest period (>20 min), the running speed/grade required to elicit appropriate workloads for the MICT (65% V̇o_2max_) and VICT (85% V̇o_2max_) sessions were determined. Participants then practiced “all-out” running on a specialized self-propelled treadmill (HiTrainer Pro, QC, Canada) on which the SIT protocol was performed.

#### Experimental sessions.

**Fig. 1.**
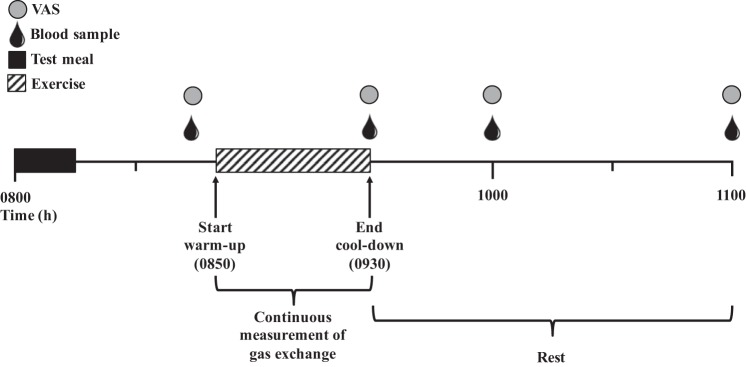
Experimental session timeline. VAS, visual analog scale.

Participants arrived at the laboratory at 0800 after an overnight fast and remained in the laboratory for the next ~3 h (Fig. 1). Upon arrival, participants were given a standardized test meal (7 kcal/kg) consisting of an appropriate amount (g) of Chocolate Chip Clif Bar (68% carbohydrates, 17% fat, 15% protein) and water (provided ad libitum throughout session). The test meal was consumed within 15 min, after which participants rested quietly while sitting for 30 min. Exercise commenced at 0850 and consisted of a 5-min standardized warm-up (3.5 mph), a 30-min running-based protocol (14 min for SIT with an additional 16 min of rest before warm-up to match protocol duration), and a 5-min cool-down (self-paced). Gas exchange (V̇o_2_ and V̇co_2_) and HR were measured continuously during exercise using the gas collection system and integrated HR monitor described previously. Upon completion of exercise (0930), participants rested quietly (reading or using a laptop) for an additional 90 min. Venous blood samples were obtained at 0845 (preexercise), 0930 h (immediately postexercise), 1000 (30 min postexercise), and 1100 (90 min postexercise). Appetite perceptions were assessed at the same blood sampling time points. Identical procedures were followed during the CTRL session with the exception of the exercise period (0850–0930), during which participants rested quietly.

#### Exercise protocols.

The MICT and VICT protocols were performed on a motorized treadmill (4Front, Woodway, WI) and consisted of 30 min of continuous running at a target workload of 65% and 85% V̇o_2max_, respectively. Participants began running at the predetermined work rate corresponding to the target intensity, and V̇o_2_ was continuously monitored to adjust work rate as needed to maintain intensity (using speed/grade adjustments). The SIT protocol was performed on a self-propelled sprint treadmill (HiTrainer Pro, QC, Canada) and consisted of four, 30-s “all out” running efforts interspersed with 4-min rest periods. Participants were instructed to exert maximal effort for the entire duration of each sprint, and strong verbal encouragement was provided throughout.

#### Blood processing and analysis.

Blood samples were collected by venipuncture from the antecubital vein, while participants were in a supine position for the measurement of acylated ghrelin, active GLP-1 (GLP-1_7–36_ and GLP-1_7–37_), total PYY, IL-6, and lactate. Two samples (3 ml whole blood each) were collected into separate prechilled Vacutainer tubes coated with K_2_ EDTA (5.4 mg) at each time point. In the first tube, 40 μl of AEBSF (25 mg/ml) per milliliter of whole blood was added to prevent degradation of acylated ghrelin. In the other tube, 10 μl of DPP-IV inhibitor and 500 KIU aprotinin per milliliter of whole blood were added to prevent inactivation of GLP-1 and ex vivo conversion of PYY_1–36_ to PYY_3–36_. All tubes were gently inverted 10 times and centrifuged at 3,000 *g* for 10 min. The plasma supernatant was then dispensed into Eppendorf tubes, while the plasma from the ghrelin Vacutainer was acidified by the addition of 100 μl of 1 M HCl per milliliter of plasma. All plasma supernatant was stored at −80°C for subsequent analysis. Commercially available enzyme-linked immunosorbent assay kits were used to determine plasma concentrations of acylated ghrelin (EMD Millipore, Billerica, MA), active GLP-1 (EMD Millipore), total PYY (EMD Millipore), and IL-6 (R&D Systems, Minneapolis, MN), according to the manufacturer’s instructions. All samples were run in duplicate and were batch analyzed for each participant to eliminate interassay variation. The intra-assay coefficients of variation for acylated ghrelin, active GLP-1, total PYY, and IL-6 were 6.6, 9.1, 4.3, and 4.8%, respectively. A droplet of blood was taken from one of the Vacutainer tubes and placed on a lactate strip for the measurement of blood lactate using a hand-held analyzer (Accutrend lactate; Roche Diagnostics, Mannheim, Germany).

#### Appetite perceptions and energy intake.

Appetite perceptions were assessed using Visual Analog Scales (VAS) (19) for perceptions of hunger (i.e., “How hungry do you feel?”), satisfaction (i.e., “How satisfied do you feel?”), fullness (i.e., “How full do you feel?”), and prospective food consumption (i.e., “How much do you think you can eat?”) on a 100-mm scale anchored at each end with contrasting statements (i.e., “not at all” and “extremely”). The mean values of the four appetite perceptions were used to calculate an overall appetite score after inverting the values for satisfaction and fullness (53). Free-living energy intake was recorded for a 3-day period (starting on the day before each experimental session) using self-reported dietary logs. For subsequent experimental sessions, participants were provided with a copy of their dietary intake from the day before their first experimental session and were asked to replicate that intake on the day before all subsequent sessions (2nd through 4th). A 24-h recall was conducted during follow-up interviews (on the morning of each session and within 48 h after each session) to verify the accuracy of the self-reported food intake (27). Participants were also provided detailed instructions (including a sample log) to ensure proper measurement and recording. Energy and macronutrient intake were calculated using the U.S. Department of Agriculture online nutritional database.

#### Statistical analysis.

All data were analyzed using SigmaStat for Windows (version 3.5). Because of the individual variability in absolute hormone concentrations and appetite perceptions, changes at each time point were expressed relative to each participant’s baseline values, as described previously (20). All area under the curve (AUC) calculations for blood-related parameters and appetite perceptions were performed using the trapezoid method using changes in each variable relative to baseline. One-way repeated-measures analysis of variance (ANOVA) was used to compare absolute hormone concentrations at baseline, AUC values across sessions, free-living energy intake, and exercise parameters. Two-way repeated-measures ANOVA (session × time) was used to compare differences in appetite-regulating hormones, lactate, IL-6, and appetite perceptions between experimental sessions at each time point. Tukey’s HSD tests were used for post hoc analysis where necessary. Relationships between variables were assessed using Pearson product-moment correlations. Specifically, the time points that elicited the greatest change in blood lactate and IL-6 were compared with the overall responses (i.e., AUC) for each appetite-regulating hormone and overall appetite during all experimental sessions. Significance was set at *P* < 0.05. All data are presented as means ± SD.

## RESULTS

### 

#### Participant characteristics.

Participants were 23.1 ± 3.0 yr of age with a mean V̇o_2max_ of 51.2 ± 4.4 ml·kg^−1^·min^−1^ (4.01 ± 0.27 l/min) and the following physical characteristics: height: 178.2 ± 2.7 cm; weight: 78.7 ± 8.1 kg; and BMI: 24.8 ± 2.3 kg/m^2^.

#### Exercise responses.

The MICT, VICT, and SIT protocols were completed at work rates corresponding to 2.64 ± 0.19 l/min (65.9 ± 1.9% V̇o_2max_), 3.29 ± 0.22 l/min (82.0 ± 0.8% V̇o_2max_), and 1.47 ± 0.09 l/min (36.8 ± 3.4% V̇o_2max_), respectively. Average HR was not different (*P* = 0.363) between MICT (161 ± 12 bpm) and VICT (172 ± 18 bpm), although both elicited a higher (*P* < 0.006) average HR than SIT (130 ± 13 bpm). Respiratory exchange ratio (RER) was highest (*P* < 0.001) during SIT (1.41 ± 0.07), while RER during VICT (1.04 ± 0.03) was also greater (*P* = 0.004) than MICT (0.98 ± 0.05). Energy expenditure based on V̇o_2_ (assuming 5 kcal/l O_2_) was highest (*P* < 0.001) during VICT (492.8 ± 33.4 kcal), while energy expenditure during MICT (396.1 ± 28.8 kcal) was also greater (*P* < 0.001) than SIT (132.3 ± 8.2 kcal).

#### Acylated ghrelin.

**Fig. 2.**
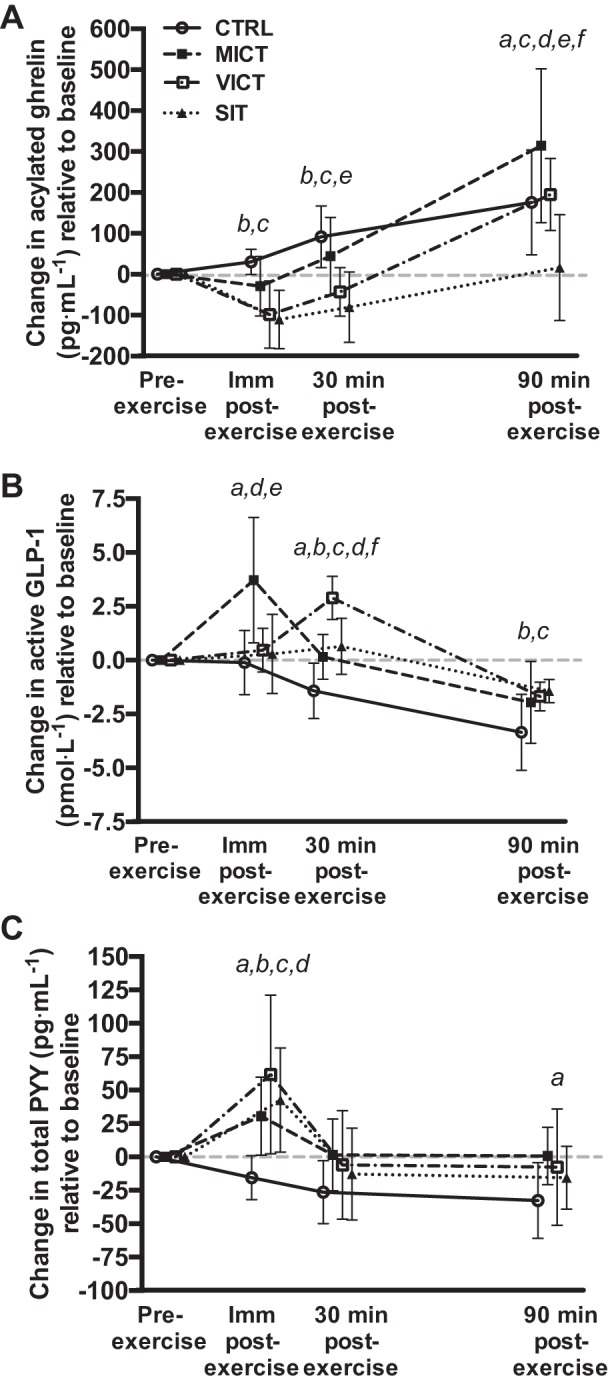
Changes in acylated ghrelin (*A*; *n* = 8), active GLP-1 (*B*; *n* = 8), and total PYY (*C*; *n* = 7) across all time points in each experimental session relative to baseline. CTRL, control; MICT, moderate-intensity continuous training; SIT, sprint interval training; VICT, vigorous-intensity continuous training. ^a^MICT significantly different from CTRL, *P* < 0.05. ^b^VICT significantly different from CTRL, *P* < 0.05. ^c^SIT significantly different from CTRL, *P* < 0.05. ^d^VICT significantly different from MICT, *P* < 0.05. ^e^SIT significantly different from MICT, *P* < 0.05. ^f^SIT significantly different from VICT, *P* < 0.05.

There were no differences (*P* > 0.636) in absolute acylated ghrelin concentrations at baseline (CTRL: 193.9 ± 114.5 pg/ml; MICT: 216.2 ± 164.6 pg/ml; VICT: 231.1 ± 185.2 pg/ml; SIT: 223.1 ± 163.2 pg/ml). Two-factor repeated-measures ANOVA revealed a significant (*P* < 0.001) interaction (session × time) for changes in acylated ghrelin relative to baseline (Fig. 2*A*). Acylated ghrelin was suppressed immediately and 30 min postexercise after VICT (*P* < 0.001) and SIT (*P* < 0.001), while MICT was not different vs. CTRL (*P* > 0.177). SIT also elicited lower acylated ghrelin concentrations at 30 min postexercise vs. MICT (*P* = 0.016) and at 90 min postexercise vs. all other sessions (*P* < 0.001). Acylated ghrelin concentrations at 90 min postexercise were not different between VICT and CTRL (*P* = 0.925), although both were lower compared with MICT (*P* < 0.003). The AUC for acylated ghrelin (Table 1) was significantly lower during SIT (*P* = 0.024) and VICT (*P* = 0.026) vs. MICT, while the acylated ghrelin AUC during SIT was also lower than CTRL (*P* = 0.020).

**Table 1.** Area under the curve values during each experimental session

	CTRL	MICT	VICT	SIT
Acylated ghrelin	175.31 ± 113.80	152.00 ± 117.40	3.80 ± 82.12^b^	−69.49 ± 146.52^a^^b^
Active GLP-1	−2.81 ± 2.31	1.46 ± 2.96#	1.62 ± 1.18^a^	−0.05 ± 2.00^a^
Total PYY	−45.83 ± 36.27	20.61 ± 39.86^a^	30.25 ± 57.70#	9.20 ± 49.08
Lactate	−0.45 ± 0.53	−0.59 ± 0.51	2.19 ± 0.88^a^^b^	8.88 ± 2.81^a^^b^^c^
IL-6	0.28 ± 0.81	1.47 ± 0.66	2.43 ± 1.06^a^^b^	1.73 ± 0.55^a^
Appetite	52.42 ± 12.69	26.20 ± 48.28	−8.16 ± 45.93^a^	−29.13 ± 45.31^a^^b^

Values are expressed as means ± SD. CTRL, control; MICT, moderate-intensity continuous training; SIT, sprint interval training; VICT, vigorous-intensity continuous training; GLP-1, glucagon-like peptide-1; PYY, peptide YY.

a Significantly different vs. CTRL (*P* < 0.05).

b Significantly different vs. MICT (*P* < 0.05).

c Significantly different vs. VICT (*P* < 0.05).

# Trend observed vs. CTRL (*P* = 0.058).

#### Active GLP-1.

There were no differences (*P* = 0.925) in absolute active GLP-1 concentrations at baseline (CTRL: 8.46 ± 2.45 pmol/l; MICT: 7.95 ± 2.06 pmol/l; VICT: 7.71 ± 2.28 pmol/l; SIT: 8.23 ± 2.54 pmol/l). Two-factor repeated-measures ANOVA revealed a significant (*P* < 0.001) interaction (session × time) for changes in active GLP-1 relative to baseline (Fig. 2*B*). MICT elicited the greatest active GLP-1 response immediately postexercise (*P* < 0.001), while VICT and SIT were both unchanged at this time point (*P* > 0.682). All three protocols increased active GLP-1 at 30 min postexercise vs. CTRL (*P* < 0.022), although this response was greatest after VICT (*P* < 0.001). Active GLP-1 remained elevated at 90 min postexercise after VICT (*P* = 0.012) and SIT (*P* = 0.003) vs. CTRL. The AUC for active GLP-1 (Table 1) was significantly greater during VICT (*P* = 0.005) and SIT (*P* = 0.017) vs. CTRL, and the difference between MICT and CTRL approached significance (*P* = 0.058).

#### Total PYY.

PYY data for one participant was excluded from the analysis due to problems with the assay (*n* = 7 for this hormone only). There were no differences (*P* > 0.432) in absolute total PYY concentrations at baseline (CTRL: 104.7 ± 61.9 pg/ml; MICT: 87.7 ± 46.2 pg/ml; VICT: 80.5 ± 48.9 pg/ml; SIT: 93.6 ± 33.7 pg/ml). Two-factor repeated-measures ANOVA revealed a significant (*P* < 0.001) interaction (session × time) for changes in total PYY concentrations relative to baseline (Fig. 2*C*). Specifically, total PYY increased immediately postexercise after all three protocols vs. CTRL (*P* < 0.001), and this response was greater after VICT compared with MICT (*P* = 0.027). There were no differences in total PYY concentrations at 30 min postexercise, although the difference between MICT and CTRL approached significance (*P* = 0.057). MICT elicited an increase at PYY at 90 min postexercise vs. CTRL (*P* = 0.016). The AUC for total PYY (Table 1) was significantly greater during MICT compared with CTRL (*P* = 0.006).

#### Lactate.

**Fig. 3.**
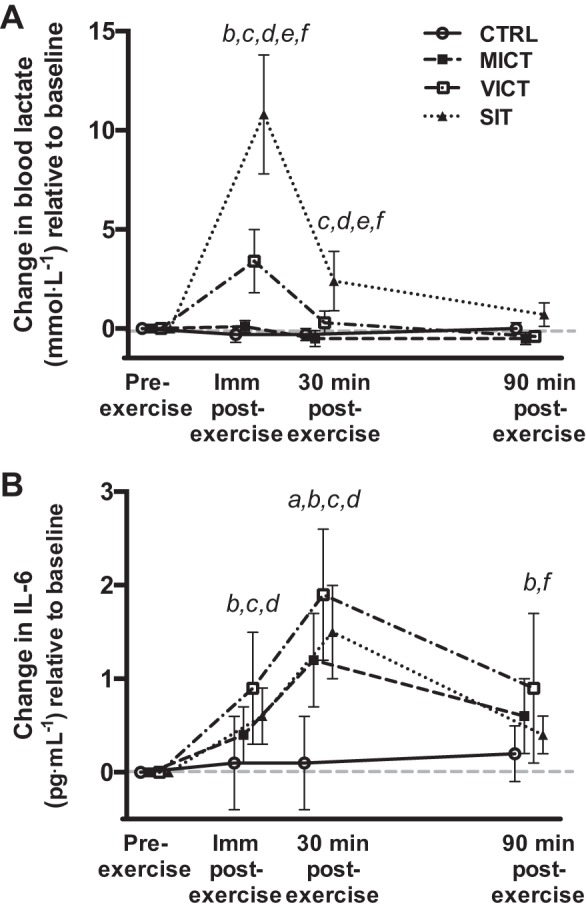
Changes in blood lactate (*A*) and IL-6 (*B*) across all time points in each experimental session relative to baseline (*n* = 8). ^a^MICT significantly different from CTRL, *P* < 0.05. ^b^VICT significantly different from CTRL, *P* < 0.05. ^c^SIT significantly different from CTRL, *P* < 0.05. ^d^VICT significantly different from MICT, *P* < 0.05. ^e^SIT significantly different from MICT, *P* < 0.05. ^f^SIT significantly different from VICT, *P* < 0.05.

There were no differences (*P* > 0.373) in absolute lactate concentrations at baseline (CTRL: 1.6 ± 0.4 mmol/l; MICT: 1.7 ± 0.6 mmol/l; VICT: 1.6 ± 0.3 mmol/l; SIT: 1.3 ± 0.7 mmol/l). Two-factor repeated-measures ANOVA revealed a significant (*P* < 0.001) interaction (session × time) for changes in lactate relative to baseline (Fig. 3*A*). SIT elicited the greatest increase in lactate immediately postexercise (*P* < 0.001), while VICT also resulted in higher blood lactate than MICT at this time point (*P* < 0.001). Lactate remained elevated at 30 min postexercise after SIT vs. all other sessions (*P* < 0.001) and returned to baseline values at 90 min postexercise (*P* > 0.430). The AUC for blood lactate (Table 1) was highest during the SIT session (*P* < 0.001), while the lactate AUC during VICT was also greater than MICT (*P* < 0.001) and CTRL (*P* = 0.001).

#### IL-6.

There were no differences (*P* = 0.301) in absolute IL-6 concentrations at baseline (CTRL: 1.61 ± 0.27 pg/ml; MICT: 1.41 ± 0.26 pg/ml; VICT: 1.57 ± 0.29 pg/ml; SIT: 1.37 ± 0.35 pg/ml). Two-factor repeated-measures ANOVA revealed a significant (*P* < 0.001) interaction (session × time) for changes in IL-6 concentrations relative to baseline (Fig. 3*B*). IL-6 increased immediately postexercise after VICT (*P* < 0.001) and SIT (*P* = 0.020) vs. CTRL, while VICT also elicited a greater response vs. MICT (*P* = 0.033). All three protocols increased IL-6 at 30 min postexercise (*P* < 0.001), with the increase being greater after VICT compared with MICT (*P* = 0.002). IL-6 concentrations remained elevated at 90 min postexercise after VICT compared with both SIT (*P* = 0.022) and CTRL (*P* = 0.002). The AUC for IL-6 (Table 1) was significantly greater during VICT (*P* = 0.018) and SIT (*P* = 0.024) vs. CTRL, while the IL-6 AUC during VICT was also greater than MICT (*P* = 0.029).

#### Appetite perceptions and energy intake.

**Fig. 4.**
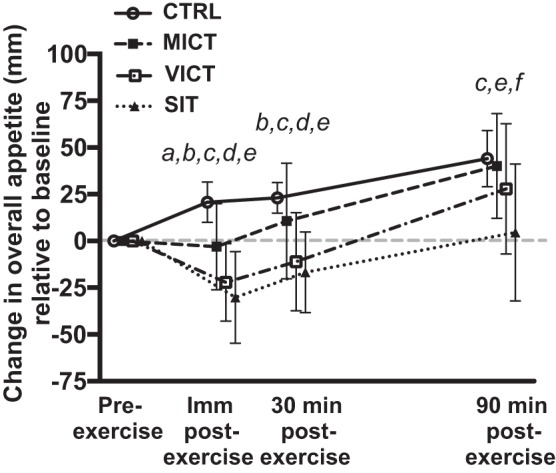
Changes in overall appetite across all time points in each experimental session relative to baseline (*n* = 8). ^a^MICT significantly different from CTRL, *P* < 0.05. ^b^VICT significantly different from CTRL, *P* < 0.05. ^c^SIT significantly different from CTRL, *P* < 0.05. ^d^VICT significantly different from MICT, *P* < 0.05. ^e^SIT significantly different from MICT, *P* < 0.05. ^f^SIT significantly different from VICT, *P* < 0.05.

**Fig. 5.**
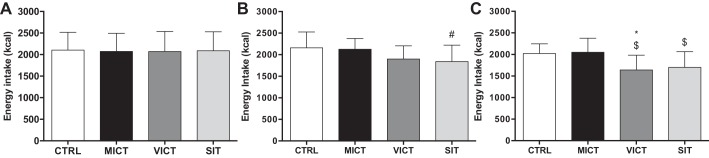
Free-living energy intake on the day before (*A*), the day of (*B*), and the day after (*C*) each experimental session (*n* = 8). *Significantly different from CTRL, *P* < 0.05. $Significantly different from MICT, *P* < 0.05. #Trend observed vs. CTRL, *P* = 0.052.

There were no differences (*P* > 0.800) in absolute values for overall appetite at baseline (CTRL: 35.0 ± 26.7 mm; MICT: 36.3 ± 29.0 mm; VICT: 36.3 ± 22.4 mm; SIT: 39.1 ± 25.7 mm). Overall appetite (Fig. 4) decreased after all three protocols immediately postexercise (*P* < 0.004), although more so after VICT (*P* = 0.026) and SIT (*P* < 0.001) compared with MICT. Appetite remained suppressed for 30 min after VICT (*P* < 0.001) and SIT (*P* < 0.001) relative to both MICT and CTRL, and for 90 min postexercise after SIT compared with all other sessions (*P* < 0.005). The AUC for overall appetite (Table 1) was significantly lower during VICT (*P* = 0.045) and SIT (*P* = 0.010) vs. CTRL, while the appetite AUC during SIT was also lower than MICT (*P* = 0.049). As intended, there were no differences (*P* > 0.801) in energy intake or macronutrient composition (CTRL: 50.6 ± 7.5% carbohydrates, 29.4 ± 5.4% fat, and 20.0 ± 5.3% protein; MICT: 47.6 ± 9.2% carbohydrates, 31.0 ± 6.6% fat, 20.9 ± 5.4% protein; VICT: 49.6 ± 8.8% carbohydrates, 28.9 ± 5.0% fat, 21.8 ± 7.0% protein; and SIT: 49.3 ± 7.0% carbohydrates, 29.7 ± 5.3% fat, 21.1 ± 5.3% protein) on the day before each experimental session (Fig. 5*A*). Energy intake on the day of the experimental session (Fig. 5*B*) was not different between sessions, although a trend suggesting reduced energy intake after SIT (*P* = 0.052 vs. CTRL) was observed. All participants ate the same breakfast on the basis of the 7 kcal/g body mass requirement for the standardized test meal (2.2 ± 0.2 bars; 147.9 ± 15.1 g; 545.8 ± 55.9 kcal) on the day of the experimental session. Energy intake was reduced on the day after the experimental session (Fig. 5*C*) following both VICT (*P* = 0.003 vs. MICT; *P* = 0.020 vs. CTRL) and SIT (*P* = 0.049 vs. MICT).

#### Correlations.

**Fig. 6.**
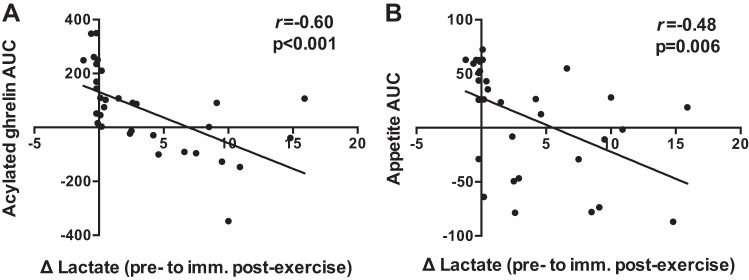
Correlations between changes in blood lactate from preexercise to immediately postexercise and the area under the curve values for acylated ghrelin (*A*) and overall appetite (*B*) during all experimental sessions (*n* = 32). *r* values represent Pearson’s correlation coefficients.

**Fig. 7.**
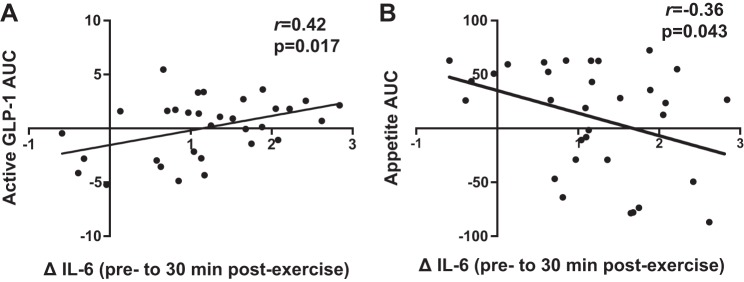
Correlations between changes in IL-6 from pre- to 30 min postexercise and the area under the curve values for active GLP-1 (*A*) and overall appetite (*B*) during all experimental sessions (*n* = 32). *r* values represent Pearson’s correlation coefficients.

The greatest change in blood lactate (Fig. 3*A*; preexercise to immediately postexercise) was significantly correlated with the AUC values for acylated ghrelin (Fig. 6*A*; *r* = −0.60, *P* < 0.001) and overall appetite (Fig. 6*B*; *r* = −0.48, *P* = 0.006) during all experimental sessions. The greatest increase in IL-6 (Fig. 3*B*; preexercise to 30 min postexercise) was significantly correlated with the AUC values for active GLP-1 (Fig. 7*A*; *r* = 0.42, *P* = 0.017) and overall appetite (Fig. 7*B*; *r* = −0.36, *P* = 0.043) during all experimental sessions. No significant relationship was observed between the postexercise increase in IL-6 and the AUC for total PYY (*r* = 0.26, *P* = 0.215). Similar correlations were observed when comparing changes at all postexercise time points between variables (data not shown).

## DISCUSSION

To the best of our knowledge, this is the first study to examine changes in appetite-related parameters and their relationship with lactate and IL-6 in response to running-based MICT, VICT, and SIT. The key findings are as follows: *1*) appetite is suppressed after acute exercise, although more so after VICT and SIT (Fig. 4), while energy intake is reduced on the day after VICT and SIT (Fig. 5); *2*) reductions in appetite are closely associated with decreases in acylated ghrelin after VICT and SIT, although SIT elicits a greater (vs. MICT) and more prolonged (vs. all other sessions) suppression of this peptide (Fig. 2); *3*) exercise intensity appears less important for active GLP-1 and total PYY, although the immediate postexercise increase in PYY is greater after VICT compared with MICT (Fig. 2); and *4*) increases in blood lactate correlate negatively with changes in acylated ghrelin and appetite (Fig. 6), while increases in IL-6 correlate positively with active GLP-1, and negatively with appetite (Fig. 7). Collectively, the observed acylated ghrelin, appetite, and energy intake responses support a clear intensity-dependent paradigm for appetite regulation following exercise. Our findings also support the potential involvement of lactate and IL-6 in the appetite-regulatory response to an acute exercise bout, although a direct/causal relationship between lactate/IL-6 and appetite-related parameters remains to be established.

The suppression of acylated ghrelin after both high-intensity protocols in our study supports a clear intensity-dependent response for this hormone. Although the effects of VICT have not been previously investigated, the majority of studies consistently demonstrating postexercise decreases in acylated ghrelin have involved more strenuous (≥70% V̇o_2max_) versions of MICT (3, 8, 9, 33, 59). At lower exercise intensities (<70% V̇o_2max_), longer-duration bouts may be necessary to suppress acylated ghrelin (13, 31, 50), although some studies have failed to show an effect, even with prolonged exercise (22, 32, 52). As such, the suppression of acylated ghrelin may be dependent on reaching an intensity threshold, particularly during short-duration exercise bouts, as highlighted by our results. Interestingly, SIT elicited a greater (vs. MICT) and more prolonged (vs. all other sessions) suppression of acylated ghrelin during the acute postexercise period of our study. In accordance with this finding, studies examining acylated ghrelin responses to repeated sprint exercise have consistently demonstrated decreases in this peptide compared with rest (13, 39, 40, 42, 52), while two of these have also reported greater reductions compared with MICT (40, 52). It is possible that the supramaximal and/or intermittent nature of SIT promotes metabolic perturbations unique to this type of exercise that influence both the magnitude and duration of the acylated ghrelin response.

The effects of exercise intensity on active GLP-1 were less pronounced. Specifically, MICT induced an increase in active GLP-1 immediately after exercise, while VICT and SIT elicited an increase at 30 min postexercise. The majority of studies examining GLP-1 responses to acute exercise have involved MICT, while only a few have measured the active forms (GLP-1_7–36_, GLP-1_7–37_) of this peptide (30, 37–39, 57, 58). The immediate postexercise GLP-1 response after MICT is in agreement with previous studies reporting rapid increases in both total and active GLP-1 following similar intensities (50–75% V̇o_2max_) of exercise (37–39, 57, 58). Increasing the intensity of MICT from 50 to 75% V̇o_2max_ does not appear to promote a further increase active GLP-1 (57), suggesting that moderate exercise intensities sufficiently stimulate its release, which is in line with our findings. Given that the initial (0–30 min postexercise) increase in GLP-1 in our study was of similar magnitude between MICT and VICT (albeit at different time points) and more pronounced compared with SIT, exercise volume (i.e., energy expenditure) may be more important for stimulating the release of this peptide (25). However, as GLP-1 concentrations remained elevated at 90 min postexercise after VICT and SIT, the overall response was similar between the three protocols (as evidenced by the similar AUC).

Total PYY increased immediately postexercise, and this effect was greater after VICT compared with MICT, which indicates a greater responsiveness of this peptide to strenuous activity. This is in line with recent work from our group demonstrating an increase in total PYY after both VICT and SIT, and a greater response after SIT compared with MICT (24). The lack of difference between SIT and MICT in the current study is likely attributable to a reduced exercise volume, as the SIT protocol that we employed previously involved two additional sprint bouts, resulting in a greater energy expenditure (24). Although we are the only group to examine PYY responses to VICT, several studies involving MICT have reported increases in total PYY (7, 13, 30, 38, 58), as well as PYY_3–36_ (14, 15, 33, 37, 57), which appear to be more pronounced after higher (≥70% V̇o_2max_) exercise intensities (7, 33, 37, 57). Although our assay detected both PYY_1–36_ and PYY_3–36_, the total PYY response that we observed is likely reflective of circulating levels of PYY_3–36_ due to the addition of DPP-IV inhibitor. Further, PYY_3–36_ comprises the majority of this peptide in circulation (fed and fasted) (4) and correlates strongly (*r* = 0.98) with measurements of total PYY (56). Studies examining PYY responses to intense-interval exercise are limited and offer mixed results, with some reporting increases (5, 15, 24) and others no changes (13, 39, 40, 42, 52). Like GLP-1, exercise duration/energy expenditure may also be important for increasing levels of this peptide, as evidenced by the MICT-induced PYY response in our study and others involving more prolonged (≥60 min) exercise protocols (7, 15, 33, 58).

All three protocols suppressed appetite immediately after exercise, although this effect persisted for 30 min after VICT and SIT, and for 90 min after SIT only. Furthermore, high-intensity exercise elicited greater appetite-suppressive effects compared with MICT at all postexercise time points. As such, exercise intensity appears to influence both the magnitude and duration of appetite suppression, with a more prolonged effect observed after supramaximal interval exercise. These findings add to previous work, demonstrating appetite suppression after strenuous (≥70% V̇o_2max_) endurance exercise (7, 9, 33). While the examination of appetite responses to intensities above 80% V̇o_2max_ has been limited to intermittent exercise, our findings extend those of Stensel’s group, who reported greater appetite-suppressive effects of submaximal (15) and supramaximal (13) interval exercise compared with MICT, although they observed compensatory increases in appetite (but not energy intake) in the hours after SIT. Although others have also reported heightened appetite perceptions after MICT that would be expected to stimulate energy intake in the hours after exercise (6, 9), we did not observe such a compensatory response after any exercise protocol within the acute experimental time frame of the current study. In fact, we observed a decrease in free living energy intake on the day after VICT and SIT, which extends previous reports of suppressed 24-h energy intake following high-intensity exercise in overweight/obese populations (52, 54), although we are the first to report this in active young males. Taken together, our data combined with previous findings supports the ability of high-intensity protocols to suppress perceptions of appetite (13, 15, 42) and facilitate reductions in energy intake (1, 52, 54).

As expected, the increase in blood lactate was intensity-dependent and greatest after SIT, providing a potential mechanism underlying the appetite-suppressive effects of high-intensity exercise. These findings are in line with those of Sim et al. (52), who reported intensity-dependent increases in blood lactate with concomitant decreases in acylated ghrelin in overweight males following submaximal and supramaximal interval cycling. Furthermore, the rapid increase in blood lactate immediately postexercise correlated negatively with the AUC values for acylated ghrelin (*r* = −0.60) and overall appetite (*r* = −0.48) in the current study. Ghrelin-producing gastric cells are highly enriched with the G protein-coupled receptor 81 (GPR81), which binds lactate, and treatment of these cells with physiological concentrations of lactate suppresses their secretory function (17). As such, the intensity-dependent reductions in acylated ghrelin following exercise may be explained by the inhibitory effects of blood lactate on these cells, which is in line with studies reporting a decrease in energy intake following peripheral lactate infusion in both humans (47) and animals (41). Alternatively, decreases in appetite and/or energy intake may also be mediated via central lactate metabolism (36), through increases in hypothalamic malonyl-CoA and subsequent changes in orexigenic and anorexigenic neuropeptides (10). Although we did not establish a causal link between blood lactate and appetite-related parameters, the aforementioned findings from animal/cell models combined with those from the current study highlight the potential involvement of lactate in the appetite-regulatory response to acute exercise.

IL-6 concentrations peaked at 30 min postexercise, with the magnitude of increase being greater after VICT compared with MICT (48). Changes in IL-6 during the postexercise period were positively correlated with the AUC values for active GLP-1 (*r* = 0.42) and peak IL-6 and GLP-1 concentrations occurred simultaneously after VICT, highlighting a possible relationship between these variables (16, 55). Although we did not observe a relationship between IL-6 and plasma total PYY in the current study, it is possible that PYY mRNA was altered in response to exercise, as previously reported in animals (16). Alternatively, a greater IL-6 response may be required to establish stronger relationships with these peptides, as the previously reported increases in GLP-1 concentrations and PYY mRNA were preceded by substantially greater (>100-fold) increases in systemic IL-6 (16). We also observed a negative correlation between changes in IL-6 during the postexercise period and the AUC values for overall appetite (*r* = −0.36), further highlighting the potential for this myokine to influence appetite and/or energy intake (2). Apart from peripheral effects, the effects of IL-6 on energy homeostasis are also mediated centrally via direct actions in key hypothalamic nuclei, as central IL-6 interacts with the same neuronal circuits by which peripheral signals exert their orexigenic/anorexigenic effects (18, 43, 44, 51). Although these findings support the role of IL-6 in appetite regulation, its proposed role as a regulator of peripheral hormone release following exercise should be further investigated in humans.

Although the current study provides valuable information regarding the effects of exercise intensity on acute appetite regulation, several limitations should be highlighted. First, given the nature of the study, it was not possible to establish a causal relationship between lactate/IL-6 and appetite-regulating hormones, such as that reported in animals/cells using direct infusion and/or knockout models. Apart from lactate and IL-6, other mechanisms (i.e., sympathetic nervous system activity, gastric blood flow/motility, circulating fuel substrates) likely also contributed to the intensity-dependent exercise effects observed in the current study (25) and should be investigated in future work. Second, the acute experimental time frame of this study may not have fully encapsulated the magnitude and/or duration of the appetite-regulatory response to exercise, limiting our ability to observe any protracted effects. Third, we acknowledge the limitations associated with self-reported energy intake (i.e., underestimation or overestimation of intake, and measurement errors), although these have shown to be of greater concern in overweight/obese populations (27). Although highly controlled laboratory conditions are no doubt ideal for isolating specific variables and examining their effects on food intake, this environment has the potential to alter an individual’s natural eating behavior. As such, we felt that it would be of greater merit to measure energy intake under free-living conditions, while trying to minimize reporting error through follow-up interviews to verify the accuracy of the self-reported dietary intake. Finally, the small sample size and the participant characteristics (active, normal weight, young males) used in this study limit the applicability of our findings to overweight/obese and/or sedentary populations. Additionally, potential sex differences in the peripheral hormonal response to exercise (26) and/or the sex-dependent expression of hypothalamic neuropeptides (49) warrant further examination of appetite control in females.

### Conclusions

Taken together, our findings support an intensity-dependent paradigm for appetite regulation following acute exercise, which appears to be closely associated with reductions in acylated ghrelin though increases in GLP-1 and PYY also contribute. Additionally, this study extends previous work in animal/cell models by providing evidence for the potential involvement of lactate and IL-6 in the appetite-regulatory response to acute exercise in humans. Nevertheless, a causal relationship between lactate/IL-6 and appetite-related parameters remains to be established and should be investigated in future work.

## GRANTS

This project was supported by an operating grant from the Natural Sciences and Engineering Research Council (NSERC; Grant RGPIN-2016-06118) to T. J. Hazell. L. K. Townsend was supported by an Ontario Graduate Scholarship.

## DISCLOSURES

No conflicts of interest, financial or otherwise, are declared by the authors.

## AUTHOR CONTRIBUTIONS

H.I., L.K.T., B.J.G., and T.J.H. conceived and designed research; H.I., L.K.T., and G.L.M. performed experiments; H.I., L.K.T., G.L.M., and P.J.M. analyzed data; H.I., L.K.T., P.J.M., B.J.G., and T.J.H. interpreted results of experiments; H.I. prepared figures; H.I. drafted manuscript; H.I., L.K.T., G.L.M., P.J.M., B.J.G., and T.J.H. edited and revised manuscript; H.I., L.K.T., G.L.M., P.J.M., B.J.G., and T.J.H. approved final version of manuscript.

## References

[1] Alkahtani SA, Byrne NM, Hills AP, King NA Interval training intensity affects energy intake compensation in obese men. Int J Sport Nutr Exerc Metab 24: 595–604, 2014. doi:10.1123/ijsnem.2013-0032. 24668572

[2] Almada C, Cataldo LR, Smalley SV, Diaz E, Serrano A, Hodgson MI, Santos JL Plasma levels of interleukin-6 and interleukin-18 after an acute physical exercise: relation with post-exercise energy intake in twins. J Physiol Biochem 69: 85–95, 2013. doi:10.1007/s13105-012-0191-x. 22810957

[3] Balaguera-Cortes L, Wallman KE, Fairchild TJ, Guelfi KJ Energy intake and appetite-related hormones following acute aerobic and resistance exercise. Appl Physiol Nutr Metab 36: 958–966, 2011. doi:10.1139/h11-121. 22111518

[4] Batterham RL, Heffron H, Kapoor S, Chivers JE, Chandarana K, Herzog H, Le Roux CW, Thomas EL, Bell JD, Withers DJ Critical role for peptide YY in protein-mediated satiation and body-weight regulation. Cell Metab 4: 223–233, 2006. doi:10.1016/j.cmet.2006.08.001. 16950139

[5] Beaulieu K, Olver TD, Abbott KC, Lemon PWR Energy intake over 2 days is unaffected by acute sprint interval exercise despite increased appetite and energy expenditure. Appl Physiol Nutr Metab 40: 79–86, 2015. doi:10.1139/apnm-2014-0229. 25494974

[6] Blundell JE, Stubbs RJ, Hughes DA, Whybrow S, King NA Cross talk between physical activity and appetite control: does physical activity stimulate appetite? Proc Nutr Soc 62: 651–661, 2003. doi:10.1079/PNS2003286. 14692601

[7] Broom DR, Batterham RL, King JA, Stensel DJ Influence of resistance and aerobic exercise on hunger, circulating levels of acylated ghrelin, and peptide YY in healthy males. Am J Physiol Regul Integr Comp Physiol 296: R29–R35, 2009. doi:10.1152/ajpregu.90706.2008. 18987287

[8] Broom DR, Miyashita M, Wasse LK, Pulsford R, King JA, Thackray AE, Stensel DJ Acute effect of exercise intensity and duration on acylated ghrelin and hunger in men. J Endocrinol 232: 411–422, 2017. doi:10.1530/JOE-16-0561. 27999089

[9] Broom DR, Stensel DJ, Bishop NC, Burns SF, Miyashita M Exercise-induced suppression of acylated ghrelin in humans. J Appl Physiol (1985) 102: 2165–2171, 2007. doi:10.1152/japplphysiol.00759.2006. 17347386

[10] Cha SH, Lane MD Central lactate metabolism suppresses food intake via the hypothalamic AMP kinase/malonyl-CoA signaling pathway. Biochem Biophys Res Commun 386: 212–216, 2009. doi:10.1016/j.bbrc.2009.06.017. 19523445

[11] Cullen T, Thomas AW, Webb R, Hughes MG Interleukin-6 and associated cytokine responses to an acute bout of high-intensity interval exercise: the effect of exercise intensity and volume. Appl Physiol Nutr Metab 41: 803–808, 2016. doi:10.1139/apnm-2015-0640. 27377137

[12] Cummings DE, Overduin J Gastrointestinal regulation of food intake. J Clin Invest 117: 13–23, 2007. doi:10.1172/JCI30227. 17200702 PMC1716217

[13] Deighton K, Barry R, Connon CE, Stensel DJ Appetite, gut hormone and energy intake responses to low volume sprint interval and traditional endurance exercise. Eur J Appl Physiol 113: 1147–1156, 2013. doi:10.1007/s00421-012-2535-1. 23111564

[14] Deighton K, Batterham RL, Stensel DJ Appetite and gut peptide responses to exercise and calorie restriction. The effect of modest energy deficits. Appetite 81: 52–59, 2014. doi:10.1016/j.appet.2014.06.003. 24911618

[15] Deighton K, Karra E, Batterham RL, Stensel DJ Appetite, energy intake, and PYY3-36 responses to energy-matched continuous exercise and submaximal high-intensity exercise. Appl Physiol Nutr Metab 38: 947–952, 2013. doi:10.1139/apnm-2012-0484. 23905660

[16] Ellingsgaard H, Hauselmann I, Schuler B, Habib AM, Baggio LL, Meier DT, Eppler E, Bouzakri K, Wueest S, Muller YD, Hansen AMK, Reinecke M, Konrad D, Gassmann M, Reimann F, Halban PA, Gromada J, Drucker DJ, Gribble FM, Ehses JA, Donath MY Interleukin-6 enhances insulin secretion by increasing glucagon-like peptide-1 secretion from L cells and alpha cells. Nat Med 17: 1481–1489, 2011. doi:10.1038/nm.2513. 22037645 PMC4286294

[17] Engelstoft MS, Park W-M, Sakata I, Kristensen LV, Husted AS, Osborne-Lawrence S, Piper PK, Walker AK, Pedersen MH, Nøhr MK, Pan J, Sinz CJ, Carrington PE, Akiyama TE, Jones RM, Tang C, Ahmed K, Offermanns S, Egerod KL, Zigman JM, Schwartz TW Seven transmembrane G protein-coupled receptor repertoire of gastric ghrelin cells. Mol Metab 2: 376–392, 2013. doi:10.1016/j.molmet.2013.08.006. 24327954 PMC3854997

[18] Ferrer B, Navia B, Giralt M, Comes G, Carrasco J, Molinero A, Quintana A, Señarís RM, Hidalgo J Muscle-specific interleukin-6 deletion influences body weight and body fat in a sex-dependent manner. Brain Behav Immun 40: 121–130, 2014. doi:10.1016/j.bbi.2014.03.001. 24632224

[19] Flint A, Raben A, Blundell JE, Astrup A Reproducibility, power and validity of visual analogue scales in assessment of appetite sensations in single test meal studies. Int J Obes Relat Metab Disord 24: 38–48, 2000. doi:10.1038/sj.ijo.0801083. 10702749

[20] Gibbons C, Caudwell P, Finlayson G, Webb D-L, Hellström PM, Näslund E, Blundell JE Comparison of postprandial profiles of ghrelin, active GLP-1, and total PYY to meals varying in fat and carbohydrate and their association with hunger and the phases of satiety. J Clin Endocrinol Metab 98: E847–E855, 2013. doi:10.1210/jc.2012-3835. 23509106

[21] Goodwin ML, Harris JE, Hernández A, Gladden LB Blood lactate measurements and analysis during exercise: a guide for clinicians. J Diabetes Sci Technol 1: 558–569, 2007. doi:10.1177/193229680700100414. 19885119 PMC2769631

[22] Hagobian TA, Sharoff CG, Stephens BR, Wade GN, Silva JE, Chipkin SR, Braun B Effects of exercise on energy-regulating hormones and appetite in men and women. Am J Physiol Regul Integr Comp Physiol 296: R233–R242, 2009. doi:10.1152/ajpregu.90671.2008. 19073905 PMC2643988

[23] Harrold JA, Dovey TM, Blundell JE, Halford JCG CNS regulation of appetite. Neuropharmacology 63: 3–17, 2012. doi:10.1016/j.neuropharm.2012.01.007. 22313528

[24] Hazell TJ, Islam H, Hallworth JR, Copeland JL Total PYY and GLP-1 responses to submaximal continuous and supramaximal sprint interval cycling in men. Appetite 108: 238–244, 2017. doi:10.1016/j.appet.2016.10.006. 27721013

[25] Hazell TJ, Islam H, Townsend LK, Schmale MS, Copeland JL Effects of exercise intensity on plasma concentrations of appetite-regulating hormones: Potential mechanisms. Appetite 98: 80–88, 2016. doi:10.1016/j.appet.2015.12.016. 26721721

[26] Hazell TJ, Townsend LK, Hallworth JR, Doan J, Copeland JL Sex differences in the response of total PYY and GLP-1 to moderate-intensity continuous and sprint interval cycling exercise. Eur J Appl Physiol 117: 431–440, 2017. doi:10.1007/s00421-017-3547-7. 28154977

[27] Hise ME, Sullivan DK, Jacobsen DJ, Johnson SL, Donnelly JE Validation of energy intake measurements determined from observer-recorded food records and recall methods compared with the doubly labeled water method in overweight and obese individuals. Am J Clin Nutr 75: 263–267, 2002. 11815316 10.1093/ajcn/75.2.263

[28] Holst JJ The physiology of glucagon-like peptide 1. Physiol Rev 87: 1409–1439, 2007. doi:10.1152/physrev.00034.2006. 17928588

[29] Hussain SS, Bloom SR The regulation of food intake by the gut-brain axis: implications for obesity. Int J Obes 37: 625–633, 2013. doi:10.1038/ijo.2012.93. 22710925

[30] Kawano H, Mineta M, Asaka M, Miyashita M, Numao S, Gando Y, Ando T, Sakamoto S, Higuchi M Effects of different modes of exercise on appetite and appetite-regulating hormones. Appetite 66: 26–33, 2013. doi:10.1016/j.appet.2013.01.017. 23402716

[31] King JA, Miyashita M, Wasse LK, Stensel DJ Influence of prolonged treadmill running on appetite, energy intake and circulating concentrations of acylated ghrelin. Appetite 54: 492–498, 2010. doi:10.1016/j.appet.2010.02.002. 20152871

[32] King JA, Wasse LK, Broom DR, Stensel DJ Influence of brisk walking on appetite, energy intake, and plasma acylated ghrelin. Med Sci Sports Exerc 42: 485–492, 2010. doi:10.1249/MSS.0b013e3181ba10c4. 19952806

[33] King JA, Wasse LK, Ewens J, Crystallis K, Emmanuel J, Batterham RL, Stensel DJ Differential acylated ghrelin, peptide YY3-36, appetite, and food intake responses to equivalent energy deficits created by exercise and food restriction. J Clin Endocrinol Metab 96: 1114–1121, 2011. doi:10.1210/jc.2010-2735. 21270331

[34] King NA, Burley VJ, Blundell JE Exercise-induced suppression of appetite: effects on food intake and implications for energy balance. Eur J Clin Nutr 48: 715–724, 1994. 7835326

[35] Kojima M, Kangawa K Ghrelin: structure and function. Physiol Rev 85: 495–522, 2005. doi:10.1152/physrev.00012.2004. 15788704

[36] Lam CKL, Chari M, Wang PYT, Lam TKT Central lactate metabolism regulates food intake. Am J Physiol Endocrinol Metab 295: E491–E496, 2008. doi:10.1152/ajpendo.90481.2008. 18577696

[37] Larson-Meyer DE, Palm S, Bansal A, Austin KJ, Hart AM, Alexander BM Influence of running and walking on hormonal regulators of appetite in women. J Obes 2012: 730409–730409, 2012. doi:10.1155/2012/730409. 22619704 PMC3350972

[38] Martins C, Morgan LM, Bloom SR, Robertson MD Effects of exercise on gut peptides, energy intake and appetite. J Endocrinol 193: 251–258, 2007. doi:10.1677/JOE-06-0030. 17470516

[39] Martins C, Stensvold D, Finlayson G, Holst J, Wisloff U, Kulseng B, Morgan L, King NA Effect of moderate- and high-intensity acute exercise on appetite in obese individuals. Med Sci Sports Exerc 47: 40–48, 2015. doi:10.1249/MSS.0000000000000372. 24824772

[40] Metcalfe RS, Koumanov F, Ruffino JS, Stokes KA, Holman GD, Thompson D, Vollaard NBJ Physiological and molecular responses to an acute bout of reduced-exertion high-intensity interval training (REHIT). Eur J Appl Physiol 115: 2321–2334, 2015. doi:10.1007/s00421-015-3217-6. 26156806

[41] Nagase H, Bray GA, York DA Effects of pyruvate and lactate on food intake in rat strains sensitive and resistant to dietary obesity. Physiol Behav 59: 555–560, 1996. doi:10.1016/0031-9384(95)02109-4. 8700960

[42] Panissa VLG, Julio UF, Hardt F, Kurashima C, Lira FS, Takito MY, Franchini E Effect of exercise intensity and mode on acute appetite control in men and women. Appl Physiol Nutr Metab 41: 1–9, 2016. doi:10.1139/apnm-2016-0172. 27704908

[43] Pazos P, Lima L, Casanueva FF, Diéguez C, García MC Interleukin 6 deficiency modulates the hypothalamic expression of energy balance regulating peptides during pregnancy in mice. PLoS One 8: e72339, 2013. doi:10.1371/journal.pone.0072339. 24015235 PMC3756067

[44] Schéle E, Benrick A, Grahnemo L, Egecioglu E, Anesten F, Pálsdóttir V, Jansson JO Inter-relation between interleukin (IL)-1, IL-6 and body fat regulating circuits of the hypothalamic arcuate nucleus. J Neuroendocrinol 25: 580–589, 2013. doi:10.1111/jne.12033. 23414303

[45] Schubert MM, Desbrow B, Sabapathy S, Leveritt M Acute exercise and subsequent energy intake. A meta-analysis. Appetite 63: 92–104, 2013. doi:10.1016/j.appet.2012.12.010. 23274127

[46] Schubert MM, Sabapathy S, Leveritt M, Desbrow B Acute exercise and hormones related to appetite regulation: a meta-analysis. Sports Med 44: 387–403, 2014. doi:10.1007/s40279-013-0120-3. 24174308

[47] Schultes B, Schmid SM, Wilms B, Jauch-Chara K, Oltmanns KM, Hallschmid M Lactate infusion during euglycemia but not hypoglycemia reduces subsequent food intake in healthy men. Appetite 58: 818–821, 2012. doi:10.1016/j.appet.2012.01.022. 22314041

[48] Scott JPR, Sale C, Greeves JP, Casey A, Dutton J, Fraser WD Effect of exercise intensity on the cytokine response to an acute bout of running. Med Sci Sports Exerc 43: 2297–2306, 2011. doi:10.1249/MSS.0b013e31822113a9. 21552156

[49] Señarís RM, Trujillo ML, Navia B, Comes G, Ferrer B, Giralt M, Hidalgo J Interleukin-6 regulates the expression of hypothalamic neuropeptides involved in body weight in a gender-dependent way. J Neuroendocrinol 23: 675–686, 2011. doi:10.1111/j.1365-2826.2011.02158.x. 21564350

[50] Shiiya T, Ueno H, Toshinai K, Kawagoe T, Naito S, Tobina T, Nishida Y, Shindo M, Kangawa K, Tanaka H, Nakazato M Significant lowering of plasma ghrelin but not des-acyl ghrelin in response to acute exercise in men. Endocr J 58: 335–342, 2011. doi:10.1507/endocrj.K11E-021. 21436599

[51] Shirazi R, Palsdottir V, Collander J, Anesten F, Vogel H, Langlet F, Jaschke A, Schürmann A, Prévot V, Shao R, Jansson J-O, Skibicka KP Glucagon-like peptide 1 receptor induced suppression of food intake, and body weight is mediated by central IL-1 and IL-6. Proc Natl Acad Sci USA 110: 16,199–16,204, 2013. doi:10.1073/pnas.1306799110. 24048027 PMC3791711

[52] Sim AY, Wallman KE, Fairchild TJ, Guelfi KJ High-intensity intermittent exercise attenuates ad-libitum energy intake. Int J Obes 38: 417–422, 2014. doi:10.1038/ijo.2013.102. 23835594

[53] Stubbs RJ, Hughes DA, Johnstone AM, Rowley E, Reid C, Elia M, Stratton R, Delargy H, King N, Blundell JE The use of visual analogue scales to assess motivation to eat in human subjects: a review of their reliability and validity with an evaluation of new hand-held computerized systems for temporal tracking of appetite ratings. Br J Nutr 84: 405–415, 2000. doi:10.1017/S0007114500001719. 11103211

[54] Thivel D, Isacco L, Montaurier C, Boirie Y, Duché P, Morio B The 24-h energy intake of obese adolescents is spontaneously reduced after intensive exercise: a randomized controlled trial in calorimetric chambers. PLoS One 7: e29840, 2012. doi:10.1371/journal.pone.0029840. 22272251 PMC3260158

[55] Timper K, Dalmas E, Dror E, Rütti S, Thienel C, Sauter NS, Bouzakri K, Bédat B, Pattou F, Kerr-Conte J, Böni-Schnetzler M, Donath MY Glucose-dependent insulinotropic peptide stimulates glucagon-like peptide 1 production by pancreatic islets via interleukin 6, produced by α cells. Gastroenterology 151: 165–179, 2016. doi:10.1053/j.gastro.2016.03.003. 26971825

[56] Tsilchorozidou T, Batterham RL, Conway GS Metformin increases fasting plasma peptide tyrosine tyrosine (PYY) in women with polycystic ovarian syndrome (PCOS). Clin Endocrinol (Oxf) 69: 936–942, 2008. doi:10.1111/j.1365-2265.2008.03285.x. 18435831

[57] Ueda S-Y, Yoshikawa T, Katsura Y, Usui T, Fujimoto S Comparable effects of moderate intensity exercise on changes in anorectic gut hormone levels and energy intake to high intensity exercise. J Endocrinol 203: 357–364, 2009. doi:10.1677/JOE-09-0190. 19737911

[58] Ueda S-Y, Yoshikawa T, Katsura Y, Usui T, Nakao H, Fujimoto S Changes in gut hormone levels and negative energy balance during aerobic exercise in obese young males. J Endocrinol 201: 151–159, 2009. doi:10.1677/JOE-08-0500. 19158129

[59] Wasse LK, Sunderland C, King JA, Miyashita M, Stensel DJ The influence of vigorous running and cycling exercise on hunger perceptions and plasma acylated ghrelin concentrations in lean young men. Appl Physiol Nutr Metab 38: 1–6, 2013. doi:10.1139/apnm-2012-0154. 23368821

[60] Yang J, Brown MS, Liang G, Grishin NV, Goldstein JL Identification of the acyltransferase that octanoylates ghrelin, an appetite-stimulating peptide hormone. Cell 132: 387–396, 2008. doi:10.1016/j.cell.2008.01.017. 18267071

